# Insights into the recent advances in nano-bioremediation of pesticides from the contaminated soil

**DOI:** 10.3389/fmicb.2022.982611

**Published:** 2022-10-19

**Authors:** Yashpal Singh, Mumtesh Kumar Saxena

**Affiliations:** Department of Animal Genetics and Breeding, College of Veterinary and Animal Sciences, G.B. Pant University of Agriculture and Technology, Pantnagar, Uttarakhand, India

**Keywords:** pesticides, bioremediation, nano-bioremediation, microbial degradation, environment

## Abstract

In the present scenario, the uncontrolled and irrational use of pesticides is affecting the environment, agriculture and livelihood worldwide. The excessive application of pesticides for better production of crops and to maintain sufficient food production is leading to cause many serious environmental issues such as soil pollution, water pollution and also affecting the food chain. The efficient management of pesticide use and remediation of pesticide-contaminated soil is one of the most significant challenges to overcome. The efficiency of the current methods of biodegradation of pesticides using different microbes and enzymes depends on the various physical and chemical conditions of the soil and they have certain limitations. Hence, a novel strategy is the need of the hour to safeguard the ecosystem from the serious environmental hazard. In recent years, the application of nanomaterials has drawn attention in many areas due to their unique properties of small size and increased surface area. Nanotechnology is considered to be a promising and effective technology in various bioremediation processes and provides many significant benefits for improving the environmental technologies using nanomaterials with efficient performance. The present article focuses on and discusses the role, application and importance of nano-bioremediation of pesticides and toxic pollutants to explore the potential of nanomaterials in the bioremediation of hazardous compounds from the environment.

## Introduction

**Figure 1:**
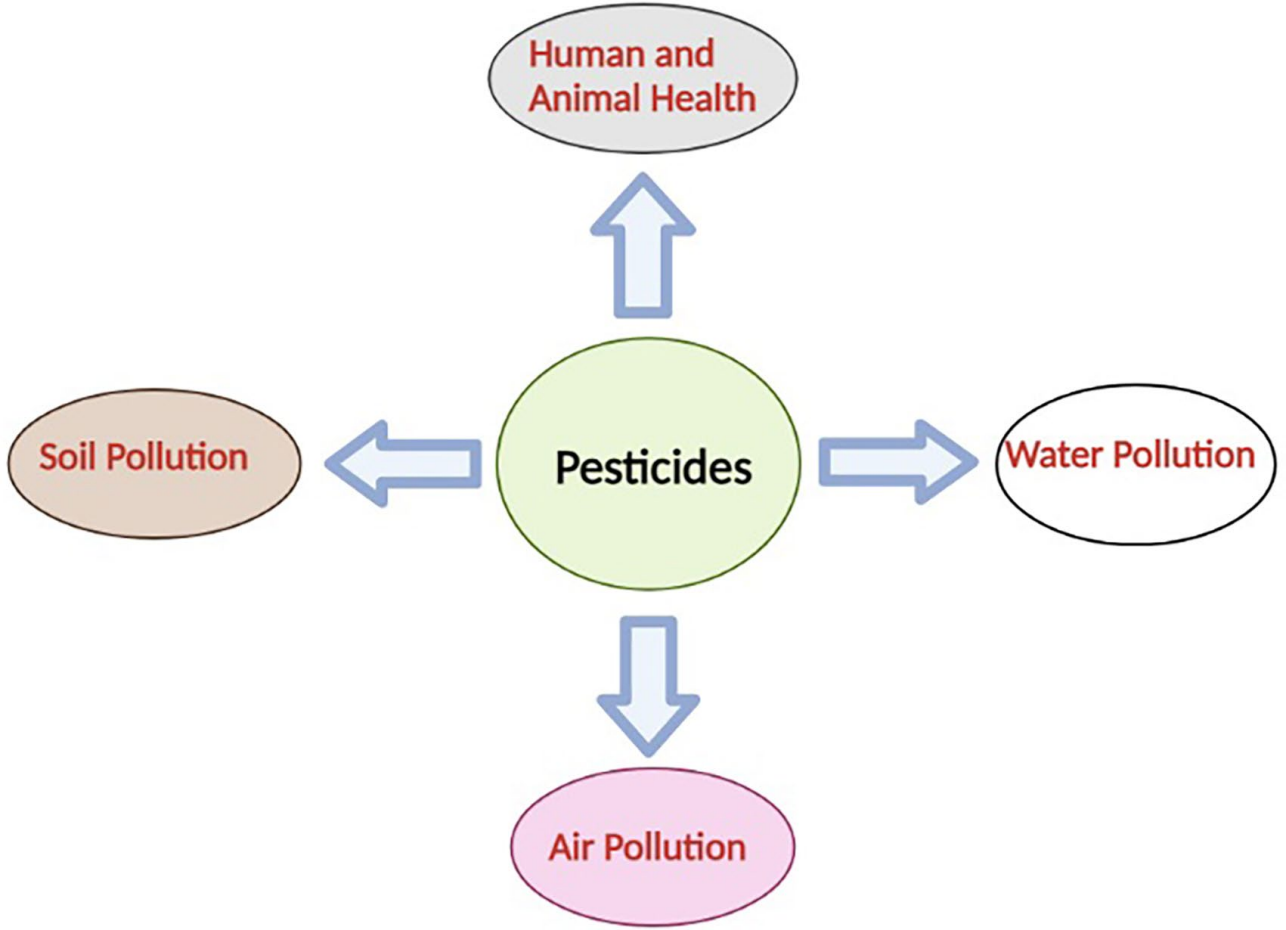
Schematic representation of the effects of pesticides on environment.

The use of pesticides increased in Indian agriculture with the green revolution between years 1967 and 1972. The use of pesticides played an important role in increasing crop production, but on the other hand, it has raised several serious issues related to human and animal health. Pesticides have been used for protection from pests but the fact is that only 1% of pesticides used could target the pests and the remaining cause contamination of soil, water and air (Sun et al., 2018; Figure 1).

Pesticides used for crop protection not only integrate into the food chain but also affect the soil health by affecting the soil microbiome and its enzyme activity (Degeronimo, 2015). Moreover, ~40% of applied pesticides are converted into transformed products, which may remain in the soil for a longer period, even for a decade (Erinle et al., 2016). These transformed products contaminated groundwater *via* leaching (Robinson and Piatt, 2019). Pesticide residues entered into the food chain affect human health by targeting several organs for example they affect the endocrine system like the thyroid gland (Huang H.S. et al., 2017), cause neurological disorders (Schmidt et al., 2017), have direct cytotoxic effects (Erdoğmuş et al., 2015) and even may increase the rate of mutations in human beings (Teodoro et al., 2019).

Chlorpyrifos (Pesticide of the organophosphate group) has been reported to cause a reduction in children’s intelligence quotients (Sun et al., 2018). Pesticide residues also affect animal health and productivity. In human beings, pesticides cause health problems while in animals also, various disease conditions like cancer, immunosuppression (Nicolopoulou-Stamati et al., 2016), birth defects, hepatic and nephrotoxicity (Choudhary et al., 2018) have been reported in the farm as well as in wild animals. As pesticides target the endocrine system, several reproductive and fertility problems have been encountered in farm animals (Marlatt et al., 2022). Pesticides not only affect the female reproductive system (Ramakrishna et al., 2022) but also are detrimental to the male reproductive system, causing toxicity to the sperm plasma membrane (Torres-Badia et al., 2022). Pesticides have deleterious effects on the biodiversity of invertebrates, mainly insects have been observed in many countries in the last few decades (Vogel, 2017). Due to the indiscriminate use of pesticides, agricultural land used by winter migrating birds is reduced and resulting in a significant reduction in the bird population. Some pesticides like imidacloprid reduced the reproductive capacity and the survival rate of birds like the white crowned sparrow (*Zonotrichia leucophrys*; Eng et al., 2017). Adverse effects of pesticides like neonicotinoids were observed in bees, bumble bees and other useful insects (Phelps et al., 2020). Even the bats were affected due to an omnivorous diet and exposed to pesticide residual contamination (Oliveira et al., 2020). Due to a reduction in the population of bees, birds and beneficial insects, pesticides have made a tremendous economic impact on the environment (Ali et al., 2021) and according to a few estimates, this loss may be 100 times more than the money spent on the conservation of the global environment and biodiversity (Organization for Economic Co-operation and Development (OECD), 2019). Around 8,000 species of insects are at the risk of extinction and in some countries like Germany, the insect population has been reduced by 75% in the last three decades (Hallmann et al., 2017). Pesticides not only affected the natural habitats and biodiversity of places where they were used, but as an effect of wind and evaporation, they could reach the atmosphere and contaminate the sites which were located far away (De Neri, 2020). Various studies have concluded that the use of pesticides for a longer period may affect soil health by interacting with the microflora, microfauna, and macrofauna of the soil (Dahiya et al., 2022).

Keeping these facts in mind, essential measures should be taken for the remediation of pesticides from soil and the environment. In this article, we have focused and discussed the significant.

The objective of this review article is to highlight the importance, impact and significant applications of the nanomaterials in the bioremediation process, for the effective remediation of toxic pollutants such as pesticides and heavy metals from the environment.

## Techniques of remediation

The soil has its capacity for degradation of compounds used in the pesticides up to a certain extent but a high concentration of these compounds is toxic for the soil microflora involved in the bioremediation and therefore needs necessary interventions in this area (Cheng et al., 2016a). Several techniques based on physical, chemical and physicochemical principles have been used for the bioremediation of the soil (Baldissarelli et al., 2019).

## Physiochemical processes of bioremediation

### Advance oxidative process

Technique of advanced oxidative process has been used as a pretreatment or treatment technique. This technique works on the principle of oxidation of polluting compounds into water, carbon dioxide and inert compounds. Ambient temperature and pressure are the pre-requisites for this technique (Cheng et al., 2016b). Though this technique has shown promising potential, most of the studies have been conducted in laboratory conditions and need further modification for scale-up in field conditions. Economic aspects of the application of this technique are also yet to be studied (Morillo and Villaverde, 2017). There are several variations of an advanced oxidative process like Fenton’s reaction, photocatalysis, plasma oxidation and ozonization, which have been tested for soil bioremediation.

### Fenton process

This process is based on the oxidation of iron ions (Fe^2+^) in a medium containing hydrogen peroxide to produce a reactive hydroxyl radical. Hydroxyl radicals further oxidize the organic pollutants into less harmful compounds. Apart from hydrogen peroxide, some other reagents like permanganate (MnO_4_^−^), persulfate (S_2_O_8_^−^) and ozone have also been used but each reagent exhibited its own merits and demerits (Cao et al., 2013; Cheng et al., 2016a). This technique has a few advantages as it may be used *in situ* (onsite) or *ex situ* (offsite) as well as it is environment friendly and easy to operate (Cao et al., 2013) but the application of this technique reduces the pH of the soil (pH < 4), which may affect the soil microbiome. Fenton’s method has mainly used an aqueous media for the treatment of groundwater or wastewater (Rosas et al., 2014). For the degradation of chlorinated pesticides like DDT (Dichlorodiphenyltrichloroethane), Fenton’s method was used in combination with other oxidative systems like zero valence ion, Ethylene diamine tetraacetic acid, air (ZVI/EDTA/Air; Cao et al., 2013) with the amino ZVI/Air system (Zhou et al., 2014) and with trisodium citrate (Vicente et al., 2012). Though the variety and efficiency of Fenton’s reaction to degrade contaminants and pollutant have shown promising potential but the studies using this technique for mass soil contaminants treatment is still the same (Baldissarelli et al., 2019).

### Heterogeneous photocatalysis

In this technique, metal oxides like titanate and zinc oxide are based as photosensitizers in the photo induce process. As they have semi filled valence band structure, they cause displacement of electrons from hydroxyl radicals of pollutant compounds (Santos et al., 2015). The efficacy of this technique depends on several factors like soil morphology, surface, pH, particle size, soil depth and light intensity (Castro et al., 2016). Though the reuse of these metal oxides is laborious yet this technique has been demonstrated by some workers in their studies (Sharma et al., 2015).

### Plasma oxidation and ozonation

Plasma oxidation is a technique of production of electrons by providing energy and space for reactive molecules. This technique may be used for the oxidation of various compounds like hydrocarbons and pesticides (Cheng et al., 2016a) but the requirement of a high energy source is a limitation of this technology. Therefore, some modifications were made to this technique and with low energy consumption; technique was used for remediation of non-miscible liquid pesticides from the soil in a short reaction time (Aggelopoulos et al., 2015). This technique has been used for the remediation of various pollutants like Pentachlorophenol (Barjasteh et al., 2021), pentanitrophenol and glyphosate (Wang et al., 2014).

### Soil washing

The technique of soil washing was used as a physical method, chemical method, or a combination of the physical and chemical methods for the treatment of organic and inorganic contaminants (Usman et al., 2022). This technique was found more effective in soil having high permeability means containing a good ratio of sand and gravel (>50%; Morillo and Villaverde, 2017). Though the soil washing technique was used effectively, its application resulted in the production of wash solution containing a high concentration of diverse pollutants and xenobiotics. Soil washing was tested in a combination with a few other techniques like Fenton oxidation to increase the efficacy of soil washing. A combination of soil washing (Sodium dodecyl sulfate as surfactant) and electrolysis was tested to remove residual pesticides (Atrazine; dos Santos et al., 2015), β methyl cyclodextrin (MCD) in combination with sunflower oil was used to remove the organochlorine pesticides from the soil (Ye et al., 2014).

### Chemical extraction-solvent extraction

The technique is based on the extraction of soil contaminants by using supercritical fluid. Extraction using a solvent system like methanol has high solvency and recovery. In this method, carbon dioxide is passed through contaminated soil and assist solubilization of toxic compounds in a solvent system like methanol (Bielská et al., 2013). The solvent system is having high solvency, and may recover a wide variety of pollutants. Though the efficacy of removal of pollutants depends on several factors like the type of extraction, and soil properties (pH, organic content, etc.). In a selective extraction system, several organic compounds were extracted using carbon dioxide as an extraction fluid (Bielská et al., 2013). In an alternative method, Forero-Mendieta et al. (2012) used a combination of carbon dioxide and methanol as a solvent and co-solvent system and remove 31 pesticides like Iprodione, tetradifon, and acephate with an efficacy of more than 70%. Supercritical fluid extraction has been used in combination with dispersive liquid–liquid micro extraction (DLLM) to detect organophosphorus pesticides like Thionazin, Sulfotep, and disulfoton in the soil. The pesticide removal was reached up to 95% with supercritical CO_2_ under the conditions of 150 bar, 60°C, 10 min of static extraction and 30 min of dynamic extraction (Naeeni et al., 2011). Veterinary pharmaceutical products and fungicides were also detected and recovered from the soil by using the solvent extraction technique (Chitescu et al., 2012). A combination of soil washing techniques with solvent extraction methods could remove DDT from the soil up to 94% (Mao et al., 2013).

### Electrokinetics

Electrokinetics, electrokinetic soil processing, or electromigration involves the application of a continuous and low-intensity current between the electrodes in the soil. Electric current causes electrolysis of soil, water and makes acidic solutions close to the anode. One of the acid fronts moves from the anode to the cathode, which results in the desorption of soil contaminants (Gomes et al., 2014). This technique results in the movement of soil pollutants and their concentration in a small area (Bocos et al., 2015). This method causes minimum disturbance in the soil environment and is economically feasible. This technique involves two processes; electromigration causes the removal of polar contaminants (ions) while electroosmosis removes non-polar contaminants (dos Santos et al., 2016). This technique is mainly used in fine granulometry soils, which have low hydraulic conductivity and large specific surface area (Morillo and Villaverde, 2017). The pesticides like Molinate and Bentazone were removed from the soil by the process of Electrokinetics, molinate was removed as catholyte while bentazone was found to be present on both of the electrodes (Ribeiro et al., 2011) but for the removal of the compounds like Pentachlorophenol (PCP), electrokinetics was not found to be very efficient technique and required techniques like permeable reactive barriers for efficient removal (Li et al., 2011). Electrokinetics could efficiently remove the commonly used pesticides 2, 4 D (2,4-dichlorophenoxyacetic acid) from the soil and reduced the concentration of 2,4 D in soil up to 80% within 60 days of treatment (Risco et al., 2015). Different configurations of electrodes have been tested to increase the efficiency of electrokinetics among which the formation of one anode and six cathodes was found more efficient than one cathode and six anodes (Risco et al., 2016). Solar power energies and wind energies were used as an alternative to conventional electric energy sources for the electrode to reduce the cost of the operation (Souza et al., 2016).

Among all the physiochemical processes described advanced oxidation process especially the Fenton technique may be considered to be the best method as it may be used for the bioremediation of a wide range of contaminants including many organic contaminants, it can be used in the area of contaminants or out of it. It is environmentally friendly and requires a shorter treatment time. It can be operated easily with low operation costs (Baldissarelli et al., 2019). But even Fenton oxidation suffers from a few limitations as it causes a reduction in soil pH, oxidizes harmless organic material of the soil and immobilization Fenton technique of inorganic reactive species on the treatment wall (Cheng et al., 2016a). Alternative techniques such as biodegradation, and using microorganisms have been used for effective decontamination of soil and water.

## Chemistry and classification of pesticides

Pesticides can be classified by several means depending on the origin of the Pesticide, chemical properties, and pest controlling capacities. Based on their origin, pesticides may be classified into two groups; biopesticides and chemical pesticides. Biopesticides may further be divided into three subgroups, i.e., microbial, biochemical and plant-incorporated protectants. Chemical pesticides can be classified based on their nature (organic and inorganic) and based on ionization properties (ionic and non-ionic). In organic and inorganic based classification, inorganic pesticides are mainly mineral derivatives while organic pesticides may be divided further into four groups; synthetic, plant based, animal based and microorganism based. Synthetic organic pesticides are of three types; organophosphates (like chlorpyrifos), organochlorine (like Lindane) and carbamates (like carbaryl). Plant-based organic pesticides can be divided into two subgroups; synthetic (like Allethrins) and natural (like Nicotine). Animal-based organic pesticides can be divided into two groups; synthetic (Fish oil) and natural (dried blood). Microorganism-based organic pesticides are of three types; bacterial (*Bacillus thuringiensis*), fungal (*Pseudozyma flocculus*) and viral (*Baculovirus*; Giri et al., 2021). Based on the classification of their ionization properties, pesticides can be divided into two groups; ionic and non-ionic pesticides. Ionic pesticides are divided into four groups, cationic (like Chlormequat, Diquat), basic (like Atrazine, Cyanazine), acidic (like Laxynil, Fenae) and miscellaneous (like Cacodylic acid, Terbacil). Non-ionic pesticides can be classified into several subgroups, chlorinated HCs (like DDT, Lindane), organophosphates (Ethion, Methyl Parathion), Dinitroanilines (like Oryzalin, Nitralin), Carbanilate (like Chlorpropham, Swep, Barban), Benzonitrile (like Dichlobenil), Ester (eg methyl ester of Chloramine), Acetamides (like CCDA), Carbothioate (like Molinate), Thiocarbamates (Metam and Ferbam), Anilides (Alachlor, Propanil), Urea (cycluron) and Methyl carbamates (carbaryl dichromate, Terbutol; Giri et al., 2021).

## Bioremediation of pesticides

The term bioremediation deals with the methods of degradation of pesticides by using the metabolic capacities of microbes. In this process, natural or genetically modified microbes utilize pesticides for their metabolic activities and convert them into environmentally benign metabolites (Karimi et al., 2022). The process of bioremediation can be classified into two groups, i.e., Bio-stimulation and Bio-augmentation.

### Biostimulation

In the process of biostimulation, microbial activity is enhanced by the addition of vitamins, substrate, oxygen and other nutrients. The addition of stimulatory nutrients results in swift depletion of the available stock of inorganic nutrient and result in pesticide degradation (Giri et al., 2021). To stimulate the process of biostimulation by microbes, water soluble nutrients like sodium nitrate, potassium nitrate, and potassium hydrogen phosphate have been added to the fertilizer (Adams et al., 2015). The ratio of Nitrogen: Phosphorous is maintained between 1:5 and 1:10 for 1%–5% N by weight of pesticide for the degradation of pesticides but if the site has been contaminated with the different types of pesticides. This ratio may not be sufficient enough for the biostimulation process. Fernandes et al. (2018) used *Pseudomonas* species for the degradation of atrazine in the soil at very high concentrations. For biostimulation, citrate at 4.8 mg/g of soil was used. The addition of citrate stimulated the bioremediation process and the efficiency of removal of atrazine was found to be 79.00 to 87%.

### Bio-augmentation

Bio-augmentation involves the addition of an exogenous micro population with the specific remediation efficacy into a polluted site. The native microbes are added to the contaminated site may be on site or off site to eliminate hazardous compounds. The process of bio-augmentation has been used for the degradation of a wide range of pollutants like NH_3_, H_2_S, organic compounds etc. from the soil and water (Hassan et al., 2022). The pre-grown microbial culture enhances the microbial population at the contaminated site and reduces the clean-up time and cost of the operation (Giri et al., 2021). Wang et al. (2013) removed atrazine from highly contaminated soil (atrazine concentration 400 mg/kg soil) by using Arthrobacter sp-based bio-augmentation. The process displayed up to 90% removal of atrazine from the soil. *Bacillus cereus* was used for the removal of chlorpyrifos contamination in the soil and the average degradation was found to be 88% within 8 days of the treatment (Farhan et al., 2021). The strains of *Pseudomonas bacillus* subtilis were used in the bio-augmentation process and removed 95% of Chlorpyrifos from the soil within 15 days of treatment (Gangola et al., 2018).

Dichloro-diphenyl-trichloroethane (DDT) has been banned in many countries for its use as a pesticide in agriculture but still, in most parts of the world, the residues of DDT are present as contaminants in the soil and its detoxification is a challenging task. Some fungus like *Gloeophyllum* trabeum and *Daedalea dickinson* has been used in the degradation of pesticides from the soil (Mathur and Gehlot, 2021). A wide range of pesticides has been degraded by the process of bio-augmentation using microorganisms. Carbofuran was degraded using *Syncephalastrum racemosum* and the rate of degradation was around 75% after inoculation of culture in the soil (de Sousa Lira and Orlanda, 2020). Cypermethrin was degraded using several bacterial strains from *Bacillus, Pseudomonas, Streptomyces*, etc. and by different fungi such as *Aspergillus niger*, *Aspergillus terricola*, *Trichoderma viride* (Maqbool et al., 2016; Bhatt et al., 2019a). Lindane and Parathion were degraded by a bio-augmentation process using *Paenibacillus dendritiformis* and *Serratia marcescens*, respectively, (Jaiswal et al., 2022). Various microorganisms, which degrade different pesticides, are enlisted (Table 1).

**Table 1:** Microorganisms involved in degradation of different pesticides.

Microorganism	Pesticide	Source	References
*Pseudomonas* sp.	Cypermethrin	Soil	Tang et al. (2015)
*Bacillus cereus*	Cypermethrin	Soil	Narayanan et al. (2020)
*Sphingomonas* sp.	Allethrin	Wastewater	Bhatt et al. (2020)
*Enterobacter* sp.	Chlorpyrifos	Soil	Singh et al. (2004)
*Sphingomonas* sp.	Oxyfluorfen	Soil	Keum et al. (2008)
*Burkholderia* sp.	Fenitrothion	Soil	Hong et al. (2007)
*Acinetobacter* sp.	Chlorpyrifos	Soil	Amani et al. (2018)
*Ochrobactrum* sp.	Methyl parathion	Soil	Qiu et al. (2007)
*Bacillus pumilus*	Chlorpyrifos	Soil	Anwar et al. (2009)
*Pseudomonas putida*	Organophosphate	Soil	Li et al. (2016)
*Burkholderia gladioli*	Prophenofos	Soil	Malghani et al. (2009)
*Bacillus aryabhattai*	Chlorpyrifos	Soil	Pailan et al. (2015)
*Bacillus subtilis* FZUL-33	Acephate	Soil	Lin et al. (2016)
*Aspergillus niger*	Cypermethrin	Soil	Bhatt et al. (2020)
*Trichoderma viridae*	Cypermethrin	Soil	Maqbool et al. (2016)

## Factors influencing the bioremediation process

Bioremediation of pesticides in the soil is a complex process, which involves several interdependent interactions within the soil, soil to air, soil to water, and characteristics of the pesticides. The rate of bioremediation depends upon interdependent physiochemical and biological processes, which are regulated by several factors.

### Chemical structure of pesticides

The chemical structure of pesticides plays an important role in regulating the rate of bioremediation of pesticides. The pesticides having polar groups like C-OH, -COOH, etc. on the phenyl ring are more susceptible to microbial biodegradation in comparison to halogen or alkyl substituents. Even a minor alteration in a structural substituent may cause drastic changes in microbial susceptibility (Geed et al., 2016). In the process of bioremediation, oxidation and reduction of active functional groups result in their conversion to simple molecules like CO_2_, H_2_O, Nitrate, Phosphate and NH_3_ (Sharma, 2020). Chlorinated hydrocarbons like DDT are more resistant to bioremediation as they have low solubility in water and high absorption affinity in soil. On the other hand, compounds like 2-4D and Carbofuran can be degraded from the soil by microorganisms in a few days within the same class of pesticides; a minor group substitution may change the susceptibility of pesticides to microbial degradation (Geed et al., 2017).

### Pesticide concentration

Pesticide concentration in soil is another important factor in deciding the rate of degradation of pesticides in the soil. The biodegradation rate depends upon the residual concentration of pesticide in the soil and it follows pseudo first order kinetics (Zaranyika et al., 2020). The rate of biodegradation decreases proportionally with residual concentration of pesticides


d[P]/dt=−K[P]

where, d[P]/dt = pesticide concentration gradient with respect to time; K = biodegradation rate constant.

The half life of pesticides may vary from 10 days to 200 days. Pesticides like Inceptisol and Ultimo have half life ranging from 10.1 to 29.2 days while several pesticides like DDT, endosulfan, and atrazine have half life varying from 100 to 200 days. The residues of less biodegradable pesticides remain adsorbed on soil particles so these are not available for microbial degradation (Giri et al., 2021).

### Soil types

Soil organic content, pH, the concentration of clay material and moisture contents are the important factors, which contribute to deciding the rate of degradation of pesticides in the soil (Rasool et al., 2022). Adsorption of pesticide residues with soil particles reduces the bioavailability of pesticides for the microbes and increases the half life of the residues (Giri et al., 2021). Water is one of the most important factors, which decides the motion and diffusion of pesticide molecules for microbial-assisted biodegradation. The rate of biodegradation of pesticides is directly proportional to soil moisture content and extremely low in dry soil (Chowdhury et al., 2008). Soil aeration and oxygen level also affect the rate of pesticide degradation as few pesticides like DDT, which is fairly stable in aerobic soil but degrades slowly in submerged soils (Raffa and Chiampo, 2021). Soil temperature produces a great impact on the stability of molecular conformation of the pesticides. It affects the solubility and rate of hydrolysis of pesticides in soil samples. The optimum soil temperature of microbial degradation ranges from 20°C to 40°C as in this range of temperature microbes has maximum activity (Singh et al., 2022).

The pH of the soil also affects the rate of bioremediation of pesticides. The degradation of the pesticides depends upon the activity of the enzymes produced by the microorganism. The enzymes have a very narrow range of pH for these activities. Most of the bacterial enzymes work at soil pH between 6.5 and 7.5. Apart from the activity of microbial enzymes produced by a microorganism, pH also influences the pesticide adsorption, biotic and abiotic degradation processes (Rasool et al., 2022). The degradation of pesticides also depends upon the chemical susceptibility toward hydrolysis by acidic or basic pH of the soil (Liu et al., 2015). Soil organic content also affects the rate of microbial degradation of pesticides. Organic contents increase the rate through co-metabolism of pesticides. The organic content of the soil also acts as a source of nutrients for the soil microbes. Therefore, increases the microbial population rapidly and results in an increased rate of microbial degradation of the pesticides. The rate of bacterial-mediated biodegradation of organochloride was increased with the addition of organic carbon sources in the soil (Krohn et al., 2021).

It has been deduced that a minimum of 1% of organic content should be present for effective microbial biodegradation of pesticides (Huang et al., 2018). In the case of pesticides, which are present in low concentrations in the soil, the co-metabolism of microbes has proven an effective measure for bioremediation. The organic content of the soil contains co-substrate, which facilitates co-metabolism of the microbes (Banwart et al., 2014).

## Carriers in bioremediation

To increase the efficacy and rate of bioremediation methods of immobilization were introduced. These methods limited the mobility of microbes and their enzymes and immobilization also enhanced the viability of microbes and the catalytic functions of their enzymes. In the process of immobilization, the natural activity of microorganisms to form biofilm on the surface of various materials was explored. Immobilization not only increased the efficacy of the bioremediation but also reduced the cost of operation as it made multiple uses of biocatalysts possible. It provided a stable environment for microbes and reduced the genetic mutations in a microorganism (Mehrotra et al., 2021). Mainly five techniques have been used in the process of immobilization; these were adsorption, binding on the surface, flocculation, entrapment and encapsulation.

### Adsorption

In this process, microorganisms are adsorbed on the surface of water insoluble carriers by weak bonds. It is a simple and economic method but as there is a high probability of cells leaking from the carriers to the environment, this method is not recommended for the use in case of genetically modified microorganisms (Dzionek et al., 2016).

### Binding on a surface

In this process, the surface of the carrier is washed with buffer, which makes the surface hydrophilic. The microbes and enzymes having a negative charge bind with the surface of the carrier. In another method of binding; covalent binding, a binding agent is required and carriers are chemically activated. This method is mainly used for the binding of enzymes because binding agents may affect the viability of microbes. As the covalent binding is very strong, the leaking of molecules (enzymes) is efficiently prevented by covalent bonding (Jiang et al., 2022).

### Entrapment in the porous matrix

This method has been mainly used in microbial bioremediation. As a result of entrapment, microbial cells can move within the carrier and prevent the leaking of the cells in the environment but allow the exchange of nutrients and metabolites. In a heterogeneous carrier system, the population of microorganisms is physiologically diverse as the cells located near the surface have high metabolic activity. The entrapment method has several advantages as it is a non-toxic, economical and highly versatile method. It efficiently prevents microorganisms from environmental factors. The efficiency of the entrapment method depends on the selection of a suitable ratio of the size of pores and cell size (Mehrotra et al., 2021).

### Encapsulation

In this method, immobilized particles are separated from the environment using a semi-permeable membrane. This method provides significant protection to microbes against external environmental conditions but the limited permeability of the membrane may affect the viability of the cells (Priyanka et al., 2022).

## Materials used for bioremediation

The method to be used for the immobilization process should sustain a few properties. It should be non-toxic, economic, stable, insoluble and regenerative. Carriers to be used for adsorption and binding on the surface should have high porosity (Dzionek et al., 2016). Carriers can be classified into two groups; natural carriers and synthetic carriers. Each group can be divided into two subgroups; organic and inorganic. Natural organic carriers include alginates, chitosan, sawdust, charcoal, plant fibers, bagasse, rice husk, etc. These carriers contain many functional groups which stabilize biocatalysts (Cubitto and Gentili, 2015). Most of these materials are waste of the food industries so these are economic and biocompatible but they have a low resistance to the biodegradation and sensitivity to organic solvent (Paliwal et al., 2015). They have a very narrow pH range for their stability. Synthetic organic carriers like polypropylene, polystyrene, polyacrylonitrile, polyvinyl alcohol and polyvinyl chloride have several functional groups with diversified properties. The macromolecular structure of synthetic organic carriers may be regulated as per the desired order of functional groups in the chain. Moreover, their porosity, polarity and hydrophobic nature can also be modified. These are commercially available at economical prices. Inorganic carriers like magnetite, silica-based material, ceramics, porous glass and nanoparticles have high chemical, physical and biological resistance. The number of functional groups present on these carriers is very less and it prevents their sufficient bonding with microorganisms and catalysts. Inorganic carriers can be used in the formation of hybrid carriers by combining them with natural polymers or synthetic nanoparticles (Yunoki et al., 2014).

## Nanotechnological interventions in bioremediation

**Figure 2:**
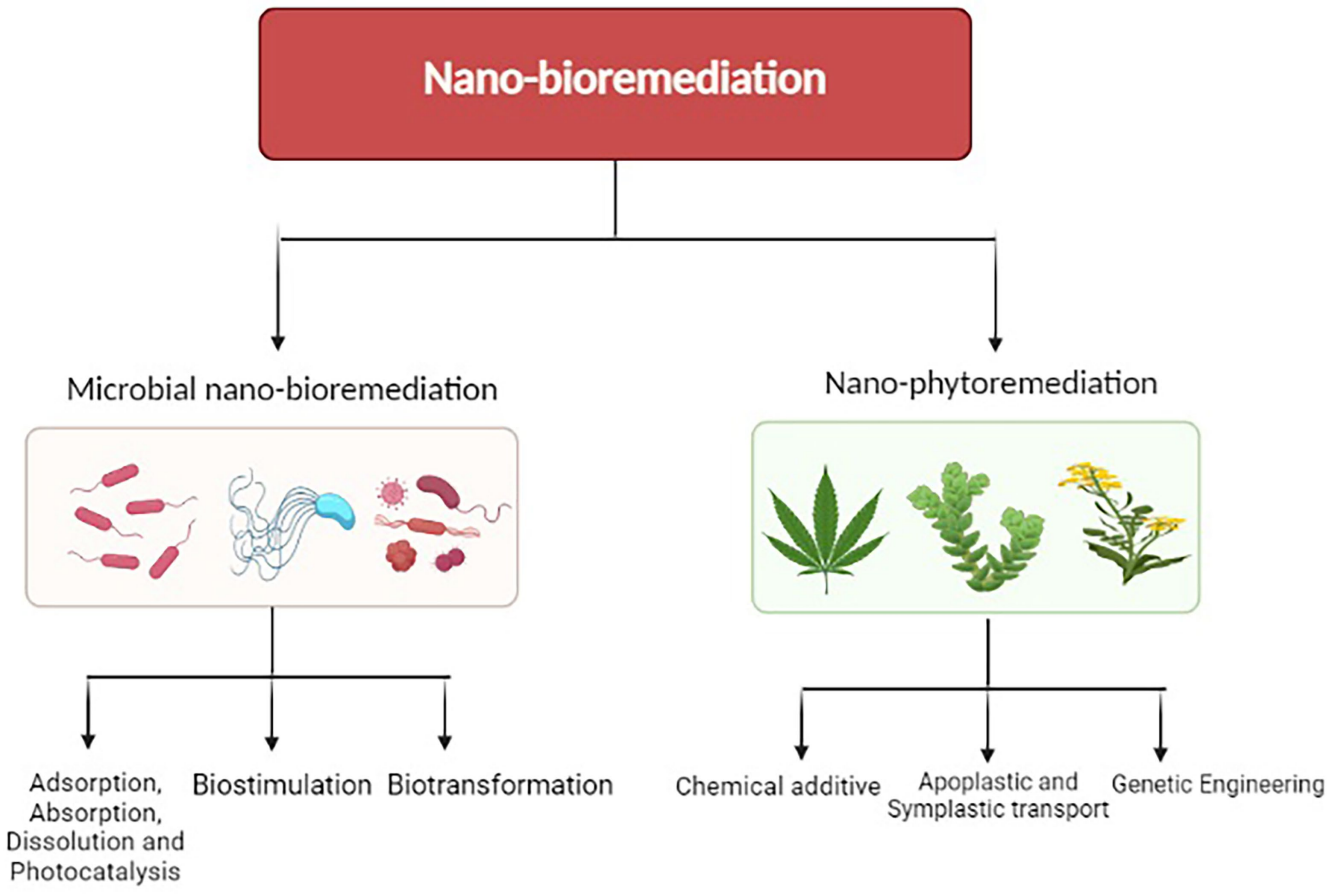
Nano-bioremediation: a promising strategy for the remediation of pesticides.

Nanotechnology is a branch of science, which deals with synthesized particles, which are very small in size (<100 nm). In the last few years, nanotechnology has been used in various fields like medicine, textiles, optics, etc. The use of nanoparticles and application of nanotechnology in agriculture was started at the beginning of the 21st century (Fraceto et al., 2016) and more than 230 nano-products have been used in various agricultural operations (Rajput et al., 2022). Nanotechnology has been integrated with the bioremediation process and termed Nano-bioremediation. Nano-bioremediation targeted cleaning of the environment by accelerating bioremediation using nanoparticles (Bhatt et al., 2021). Nano-bioremediation is further subdivided into two subgroups, i.e., nano-phytoremediation of nanoparticles with phytoremediation and microbial nano-remediation (Singh et al., 2020; Kumari et al., 2022; Figure 2).

The basic principle of nano-bioremediation is the degradation of contaminants using a catalyst as nanoparticles. As the nanoparticles are very small in size, it allows them to interact more deeply and have a large surface area as per unit mass, more numbers of nanoparticles can come into contact with the environment. This enhances the efficacy and the rate of bioremediation (Cecchin et al., 2017).

The process of bioremediation involves the use of living organisms for the remediation of pollutants. Once nanotechnology is integrated with the process of bioremediation, the interaction of nanoparticles with living organisms becomes the key factor in deciding the efficacy of nano-bioremediation. In a few cases, the interaction of nanoparticles and biotic components resulted in biocidal and proven harmful to the organisms involved in the bioremediation (Juárez-Maldonado et al., 2019). Therefore, evaluation of the interaction of nanoparticles and biotic components is the prerequisite of the nano-bioremediation process. The efficacy of nano-bioremediation can be influenced by several factors like size, shape, chemical nature of the nanoparticles, the physiological properties of the organism, pH and temperature of the soil, nature of the contaminant, etc. (Tan et al., 2018). These factors work directly or indirectly. Temperature, pH and media are crucial for the optimal development of biological organisms while the direct interaction of nanoparticles and organisms regulates various actions like dissolution, absorption and biotransformation (Kranjc and Drobne, 2019). Nano-bioremediation is a two-step process. In the first contaminants are broken down by nanoparticles to a conducive level for bioremediation and in the second step; pollutants are biodegraded (Cecchin et al., 2017).

## Nano-phytoremediation of contaminated soil

Nano-phytoremediation is a method for remediation of pollutants, and contaminants by using synthesized nanoparticles from the plants. Plants are the natural detoxifiers for the soil as they absorb diverse types of compounds and detoxify them. Phytoremediation is a Greek word, which means restore/remedy through plants (Pandya et al., 2022) but phytoremediation has a few limitations as slow remediation time and plant waste. Nanotechnology has increased the efficacy of remediation of contaminated soil and water. Organic contaminants like atrazine, molinate and chlorpyrifos have been degraded with nano-sized zerovalent irons. Enzymes encapsulated in nanoparticles increased the efficiency of bioremediation significantly (Yadav et al., 2017).

There are several factors, which affect the efficacy of nano-bioremediation. These are the physical and chemical properties of compounds, their molecular weight, solubility in the water, soil environment (pH, temperature and percentage of organic matter) and characteristics of plants (Gulzar and Mazumder, 2022). Integration of nanoparticles and phytoremediation is the most important step in nano-phytoremediation. The studies indicate that the application of nanoparticles detoxified various organic, inorganic and metal pollutants from the soil. The use of nano-zerovalent iron, and magnetite nanoparticles rapidly degraded organic pollutants from the soil (Madhura et al., 2019). Nanoparticles of TiO_2_ (n TiO_2_) and PEI-copper nanoparticles reduced the half lives of Phenanthrene and atrazine, respectively (Li et al., 2016; Kalidhasan et al., 2017). The technique of nano-phytoremediation worked for a wide range of soil pollutants ranging from heavy metals to organic compounds. The application of nanoparticles enhanced the uptake of pollutants by plants and also improved the stress tolerance capacity of the plants (Pillai and Kottekottil, 2016; Souri et al., 2017).

## Important factors in the interaction of plants and nanoparticles

Though there are several factors, which affect the uptake of nanoparticles by plants like the type and chemical composition of nanomaterial, the size of nanoparticles plays the most crucial role in the uptake of the nanoparticles (Schwab et al., 2016). The nanoparticles can be transported into the plants in two ways; Apoplastic transport (transport through xylem vessels), Symplastic transport (transport between the cytoplasm and sieve plates; Horejs, 2022).

Apart from the size of nanoparticles, the soil temperature is also an important factor as it affects the growth substances and root lengths (Ahmadpour et al., 2012). The properties of the plants also affect the efficacy of nano-bioremediation. For achieving high efficiency, a plant should have fast growth, large biomass, a well-developed root system, high toxicity tolerance limit, high accumulation capacity, a non-consumable for animals and easy for genetic manipulation. Nanoparticles should be non-toxic for plants and should have the properties to enhance germination, root-shoot elongation, enhanced phoenzyme production, increased plant growth hormone and capabilities to bind with contaminants of the soil (Sajid et al., 2015). Nano-phytoremediation technology has been used with natural as well as genetically engineered plants. Nanoparticles enhanced plant growth and their efficacy in remediation of the soil contaminants. These particles increased the production of plant growth hormone and enhanced the uptake of soil pollutants by plants (Dimkpa et al., 2012; Liu et al., 2016) Nano-zerovalent iron nanoparticles were used with plants like *Alpinia calcarata* Roscoe, *Ocimum sanctum*, *Cymbopogon citratus* and with all three plants, these particles enhanced the remediation efficacy against Endosulfan (Pillai and Kottekottil, 2016). Similarly, sialic acid nanoparticles increased the phytoremediation efficacy of Isatis cappadocica Desv for Arsenic (Souri et al., 2017). Nanoparticles cause many physiological changes in the plants, which results in increased efficacy of phytoremediation but the effectiveness and safety of the nanoparticles are decided by several factors like chemical composition, size, shape, stability, concentration, and surface coating and reactivity of the nanoparticles. The efficacy of nanoparticles may vary from plant to plant also (Varma and Khanuja, 2017; Yang et al., 2017). Nano-phytoremediation technology has few limitations such as most of the experiments have been conducted at microcosm levels so extensive studies are required. Formation of aggregation is a common phenomenon with nanoparticles so studies to modify their surfaces to enhance the sustainability of nanoparticles are essentially required and in last the toxicity of various nanoparticles for the soil and the environment needs to be evaluated.

## Microbial nano-bioremediation

**Figure 3:**
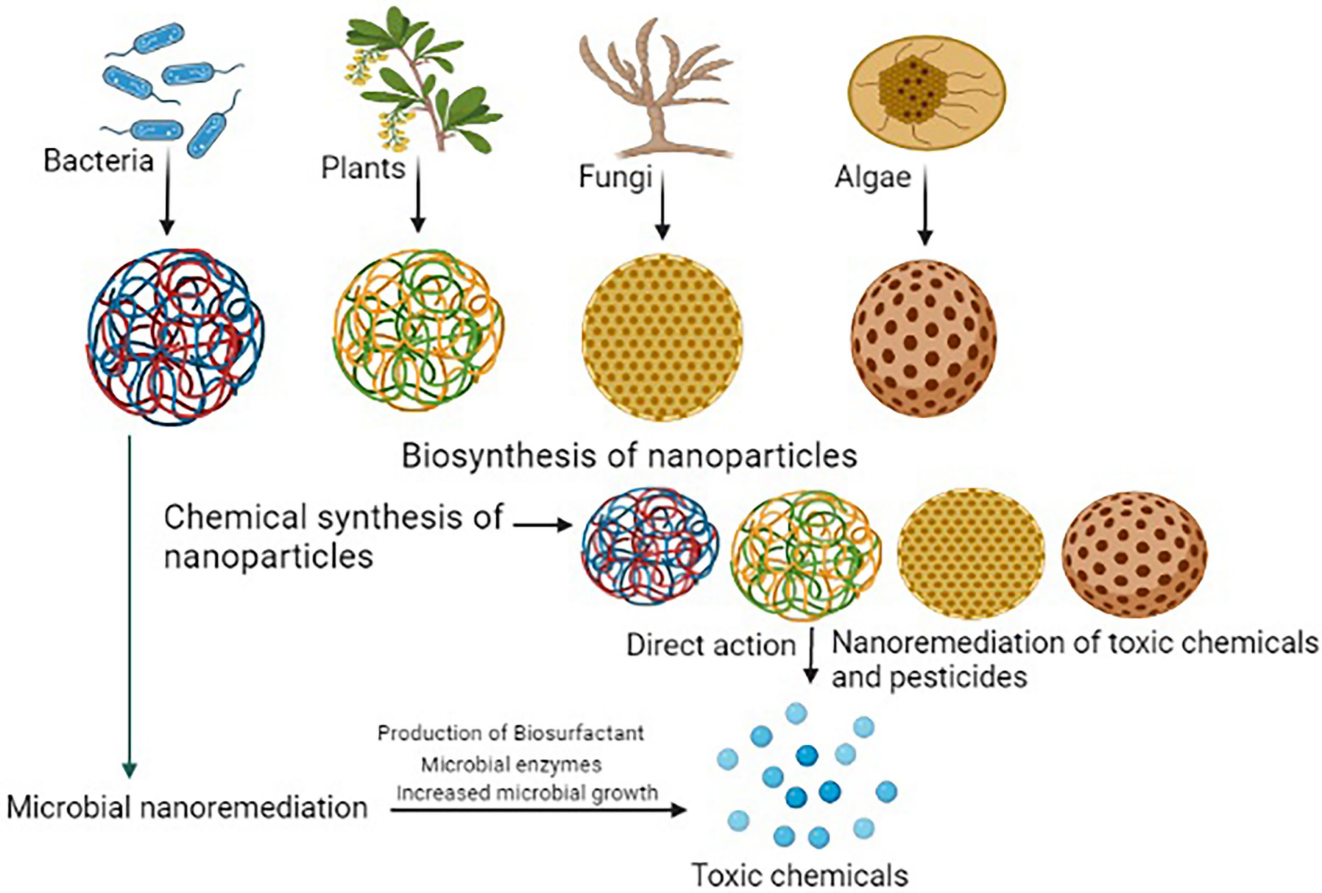
Nano-particles mediated removal of toxic pollutants from the soil.

It is the process in which nanoparticles are used with soil microbes to enhance biodegradation processes. Microorganisms can uptake the metal ions and reduce them. In this process, the metal ions are converted into nanoparticles. Microbial enzymes along with the metals form useful nanoparticles for nano-bioremediation (Pandey, 2018). Microbial nano-bioremediation is a two-phase process, which involves abiotic and biotic processes (Usman et al., 2020). In the first phase, nanoparticles enter the system and particles of pollutants undergo varieties of the processes like adsorption, absorption, dissolution and photocatalysis (Abebe et al., 2018). In the second phase, several biotic processes like biostimulation and biotransformation remove these particles from the system (Desiante et al., 2021). The second phase (biotic phase) plays a very important role in the bioremediation of pollutants. Microbial nano-bioremediation has been used for a variety of pollutants like inorganic and organic (Figure 3). Nanoparticles involved in the microbial degradation and enlisted in Table 2.

**Table 2:** Role of different nanoparticles in microbial and pesticide degradation.

**Nanoparticles**	**Function**	**References**
*Bacillus subtilis* immobilized on Ferric oxide	Degradation of azo dyes (80%)	Nadi et al. (2019)
Calcium oxide	Degradation of Glyphosate	Cabrera-Penna and Rodríguez-P’aez (2021)
Copper oxide	Dye degradation and inhibit the growth of pathogenic bacteria	Nwanya et al. (2019)
DP-ZnO NPs	Degradation of Methylene blue and eosin yellow	Rambabu et al. (2021)
Ferric oxide	Degradation of Textile effluent	Fouda et al. (2021)
*Halomonas* immobilized with magnetic nanoparticles	Removal of Palladium	Cao et al. (2020)
Iron oxide	Removal of different heavy metals from wastewater	Mahanty et al. (2020)
Magnetite	Degradation of Phenazopyridine	Gholizadeh et al. (2021)
Manganese-Titanium oxide	Degradation of Acetaldehyde	Karafas et al. (2019)
MZnO/TiO_2_-Fe_3_O_4_	Degradation of Chlorpyrifos	Saljooqi et al. (2020)
Silver oxide nanoparticles	Removal of methylene blue	Shah et al. (2019)

### Microbial nano-bioremediation for heavy metals

Heavy metals are increasing in the environment and soil due to anthropogenic activities and disturbed biogeochemical cycles. Heavy metals like Pb, As, Cd, etc. not have any well-defined function in the biological system but they have a toxic effect on the biotic component of the environment even in the low concentration (Bist and Choudhary, 2022). In acidic soil with low nutrient levels, the toxicity of heavy metals increases (Liu et al., 2018). Heavy metals generate reactive oxygen, which damages the macromolecules (proteins and nucleic acids) of microorganisms and plants (Bist and Choudhary, 2022). Immobilization of heavy metal molecules is the most common technique used for their bioremediation (Suman et al., 2018). Nanoparticles including bio-organic nanoparticles (synthesized using biological organisms) have been used in the removal of heavy metals from the soil Bio-organic nanoparticles such as silver nanoparticles produced in *Morganella psychrotolerans* have been used for the remove of heavy metals (Arif et al., 2016; Enez et al., 2018). Iron oxide nanoparticles coated with polyvinyl pyrrolidone (PVP) are used with *Halomonas* sp. (gram-negative bacteria) for the bioremediation of lead and cadmium (Alabresm et al., 2018). Magnetic nanoparticles of Fe_3_O_4_ coated with phthalic acid treated with *S. aureus* were used for bioremediation of Cu, Ni and Pb. The efficiency of these particles was 83%–89% for Cu^2+^, 99.4%–100% for Pb^2+^ and 92.6%–97.5% for Ni^2+^. It was observed that the functional group present on the microbial surface and core of nanoparticles played an important role in the removal of heavy metals (Mahmoud et al., 2016). Heavy metals resistant bacteria *B. cereus* and *L. macroides* in combination with zinc oxide were used for the remediation of Cu, Cd, Cr and Pb. It was deduced that zinc oxide nanoparticles along with *B. cereus* could remove these metals efficiently (Baragaño et al., 2020). The strain of *B. cereus* (XMCr^−6^) reduced Cr^+6^ to Cr^+3^. The reduced Cr^+3^ exhibited affinity to the bacterial cell surface and by reacting with oxygen formed Chromium oxide nanoparticles as a byproduct (Laslo et al., 2022). Selenium nanoparticles in combination with probiotic bacteria (*L. casei*) were used for cadmium-contaminated land and water treatment. The efficiency of cadmium absorption was found 65% with *L. casei*, which was significantly higher than *L. casei* alone (43%–78%; Dong et al., 2013).

In the approach of bio-organic nanoparticle synthesis, heavy metals pollutants can be used by selective microbes followed by their removal from the environment and yielding value for the waste. In this approach, Enterococcus faecelis was used for the removal and recovery of lead. The lead nanoparticles were synthesized by bacteria in extracellular and intracellular modes. The size of these nanoparticles was ~10 nm. These particles exhibited high catalytic efficiency and reduced 5.0 μmol Cr^+6^ in 12 h (Cao et al., 2020).

In anaerobic sludge, tellurium nanoparticles were synthesized by supplementation with riboflavin in the presence of *Rhodobacter* capsulates using polluted tellurite Te^4+^ oxy anions present in the wastewater (Ramos-Ruiz et al., 2016). These findings indicate that nano-bioremediation may be effectively used for the remediation of heavy metals toxicity.

### Nano-bioremediation of organic pollutants

Organic pollutants mainly persistent organic pollutants have negative impacts on human and environmental health. Therefore, the remediation of these compounds from the soil is essential. Magnetic nanoparticles in combination with *Rhodococcus engthropolis* caused the desulfurization of dibenzothiophene (DBT; Ansari et al., 2009). Bimetallic (Pd/nFe) nanoparticles were used in combination with *Sphingomonas wittichii* for bioremediation of NBR.2, 3, 7, 8-tetrachlorodibenzo-p-dioxin hydrocarbons (Bokare et al., 2012). The silica nanoparticles biofunctionalized with the lipid bilayer of *Pseudomonas* aeruginosa were used to clean up PAH(benzo[a] Pyrene). The membrane lipids of *Pseudomonas* played a role to enhance the sequestration of PAH (Wang et al., 2015). The grapheme oxide nanoparticles along with the laccase enzyme of Trametes versicolor were studied for biodegradation of PAH and were found to be effective in remediation of PAH (Patil et al., 2016). Alcaligenes faecelis in combination with iron oxide nanoparticles improved the degradation of hydrocarbon compounds of crude oil contamination (Oyewole et al., 2019). *Sphingomonas* strain NM05 has been used for the degradation of hexachlorocyclohexane (HCH). Once the strain was treated with Pd/FeO bimetallic nanoparticles, the degradation efficacy of the strain was enhanced ~2 folds (Singh R. et al., 2013). The peroskite (LaFeO_3_) nanoparticles with proteobacteria were used for the degradation of organic contaminants in marine sediments (Hung et al., 2021). Nanoparticles not only enhanced the remediation efficacy of microbes but are also used for the improvement of soil health. Silicon nanoparticles have been reported to improve the soil microflora and biomass. These particles enhanced the growth of rhizospheric microbes (Rajput et al., 2022).

## Algae mediated nano-bioremediation

Phyto-nanotechnology is an efficient, cost-effective and eco-friendly strategy, which is extensively used for the remediation of toxic compounds from the ecosystem (Gole et al., 2022). This technology involves the plant based synthesis of nanoparticles with almost no risk to the ecosystem and humans. Various types of metal nanoparticles like silver, palladium and gold have been synthesized with algae belonging to different groups such as Chlorophyceae, Cyanophyceae, Phaeophyceae and Rhodophyceae. Algae are the largest photoautotrophic group of microbes, having the potential to act as nano-machineries for the metallic nanoparticles. The fabrication of algae based nanoparticles is less time consuming process (Khanna et al., 2019). Algae have several properties like high potential of metal uptake, easy to handle and harvest, low cost, low toxicity, which make them suitable to serve as nano-factories (Sharma et al., 2015). Silver nanoparticles were produced using number of brown algae species such as *Cystophora moniliformis*, *Gelidiella acerosa* and *Padina pavonica* (Azizi et al., 2014) while other species like *Cystoseira baccata*, *Dictyota bartayresianna*, *Ecklonia cava*, and *Sargassum wightii* have been used in the fabrication of gold nanoparticles (AuNPs). Similarly *Phormidium valderianum* and *S. platensis* are also responsible for the AuNPs biosynthesis (Iravani et al., 2017). Algal species such as *Cylindrospermum stagnale*, *Spirulina platensis*, *Plectonema boryanum*, and *Microchaete diplosiphon* have been reported for the synthesis of AgNPs having varied morphologies (Husain et al., 2015). Various algae have been recognized for the remediation of toxic compounds and heavy metals from the wastewater (Goswami et al., 2021). Studies showed that the microalgae from Chlorellaceae family removed heavy metals such as lead, copper, and selenium from the wastewater (Oyebamiji et al., 2019). Microalgae have the ability to remove the toxic heavy metals from the acid mine drainage, which facilitate the inhibition of direct discharge of acid mines into the water bodies that may lead to the damage to aquatic habitat as well as create serious environmental pollution (Samal et al., 2020). The synthesis of nanoparticles and the algae mediated bioremediation belong to same process, which occur simultaneously (Dahoumane et al., 2016). Recently *Chlorella vulgaris*, a green microalgae was reported for the efficient removal of Au(I) and Au(III) complexes (He and Chen, 2014). The metal uptake potential of *Nannochloropsis oculata* was evaluated from the acid mine drainage. The result revealed that 99% of copper content was removed by *N. oculata* (Martínez-Macias et al., 2019). Furthermore, a microalga such as *Chlorella kessleri* was used for the removal of heavy metals from wastewater (Sultana et al., 2020). Studies have showed that microalgae have potential to remove heavy metals from the wastewater. However extensive research is required in this aspect to enhance the remediation efficiency and complete utilization of the biomass. Various studies have reported the wastewater treatment by the immobilization of microalgae biomass, which is considered as an effective technique for the remediation of the heavy metal (Cheng et al., 2019). The consortium of microalgae has attracted interest of the researchers to remove heavy metals for the wastewater treatment. The heavy metals such as nickel, cadmium and lead have been removed from the wastewater using the consortia of microalgae (Abdel-Razek et al., 2019).

## Fungi mediated nano-bioremediation

Fungi are the eukaryotic microorganisms, which include molds, yeasts, mildews and mushrooms (Duhan et al., 2017). Fungi act as biocatalysts and are used in bioremediation as they can survive in intense conditions as well as elevated concentration of heavy metals (Dixit et al., 2015). In green nanotechnology, nanoparticles are synthesized using fungi, which play a pivotal function in the removal of toxic compounds and organic pollutants (Singh et al., 2018). In recent times, the synthesis of metal nanoparticles from fungi has gained a big interest of researchers around the world (Sunny et al., 2022). There are several advantages of metal nanoparticles synthesized using fungi such as higher capacity of metal uptake, simple and low cost fabrication, tolerant against metals, high scalability, highly stable (Yadav et al., 2015).

Various fungus like *Fusarium*, *Verticillium*, *Penicillium*, and *Aspergillus* have been used as potential candidates for the metallic nanoparticles synthesis (Ovais et al., 2018). Studies have reported that metals like gold, silver, titanium, platinum, selenium, palladium and silica can be utilized for the fabrication of nanoparticles (Gholami-Shabani et al., 2016). Silver nanoparticles (AgNPs) synthesized from *F. oxysporum* are different in features from those synthesized with *Aspergillus fumigates* (Alves and Murray, 2022). Studies have reported the synthesis of AgNPs using *Coriolus versicolor* and *Trichoderma reesei* (Vahabi et al., 2011; Deniz et al., 2019). The gold nanoparticles (AuNPs) have been synthesized using *Cylindrocladium floridanum* fungus (Narayanan and Sakthivel, 2011). Platinum nanoparticles were synthesized using the *N. crassa* fungus and *Fusarium oxysporum* was used for fabrication of silica nanoparticles (Castro-Longoria et al., 2012). The selenium nanoparticles have been synthesized from *Mariannaea* sp. (Zhang et al., 2019). The green synthesis of the nanoparticles from different fungus has led to a significant application in the remediation of hazardous organic pollutants through the adsorption of heavy metals (Gaur et al., 2014). The kind of metal, environmental factors and fungal biomass affect the capacity of biosorption (Dhankhar and Hooda, 2011). Studies have showed the proficient adsorption, immobilization capacity and chelation activity of heavy metal ions by arbuscular mycorrhizal fungi (Upadhyaya et al., 2010). Various fungal species like *Allescheriella* sp., *Botryosphaeria rhodina* sp., *Stachybotrys* sp. exhibited the metal-binding capability (Benjamin et al., 2019). The synthesis of AgNPs from *Rhizopus oryzae* have several ecological uses like wastewater treatment (Zhang et al., 2014) and adsorption of pesticides (Das et al., 2012). Fungi like *Fusarium solani* have higher tolerance against few heavy metals like cadmium, nickel and lead, also have better capacity of nanoparticles synthesis (Rasha, 2017). The extremophilic fungi have significant ability and application in nano-bioremediation of heavy metals due to ability to survive in severe conditions, which makes them significant for the purpose of nano-bioremediation (Bahrulolum et al., 2021). The marine fungi were assessed for their potential of bioremediation as well as the ecological importance (Thatoi et al., 2013). The degradation of pentachlorophenol was observed by *Trichoderma harzianum* (Vacondio et al., 2015). The heavy metals like CdCl_2_, CuSO_4_, Pb and ZnSO_4_ could not affect the growth of *Cryptococcus* sp., a psychrophilic fungus (Singh M.P. et al., 2013).

Nanoparticles in combination with white-rot fungi (WRF) have immense potential of bioremediation (He et al., 2017). Studies have reported the remediation of toxic contaminants from wastewater and good stability of WRF-magnetic nanoparticles due to proficient immobilization. Antibiotics like sulfonamide have been reported to be degraded by *Echinodontium taxodii-*Fe_3_O_4_ nanoparticles (Shi et al., 2015). The remediation of cadmium and 2,4-dichlorophenol was achieved through immobilization of *Phanerochaete chrysosporium* along with titanium oxide nanoparticles (Chen et al., 2013) *P. chrysosporium* along with Fe_2_O_3_ nanoparticles revealed degradation of phenols (Huang Z. et al., 2017). Likewise, selenium nanoparticles in combination with *Phanerochaete chrysosporium* reported effective remediation of zinc (Espinosa-Ortiz et al., 2016). Similarly *P. chrysosporium*, with silver nanoparticles showed enhanced removal of Cd^2+^ and 2,4-dichlorophenol (Zuo et al., 2015; Huang Z. et al., 2017). Therefore, it can be concluded that nanoparticles in combination with fungi resulted in an increased rate of remediation. Hence more studies and research should be conducted for development of such effective strategies using ecological microbiology and nanotechnology.

## Conclusion

The various reports available on pesticide contamination of soil indicate that the level of pesticides in the soil is increasing day by day, which is affecting human, soil and environmental health. Hence pesticides should be used rationally, especially in the underdeveloped and developing countries where an efficient monitoring system is lacking. In case of real need, the preference should be given to the application of pesticides, which have short half life and high biodegradability. To improve soil health, crop rotation programs and the use of organic manure should be implemented more effectively. Though there are various techniques used for the remediation of soil pesticide contamination, most of these techniques have their limitations. The various experiments conducted with the integration of nanoparticles with the bioremediation process have shown promising potential but more extensive research and experimentation are required in this area. Various nanoparticles studied have enhanced the efficacy of the bioremediation but the safety of the nanoparticles for the environment and the food chain is still a matter of concern. Therefore, extensive research is required in the area of the safety analysis of nanoparticles.

## Author contributions

YS wrote the manuscript. MK conceptualized and reviewed manuscript. All authors contributed to the article and approved the submitted version.

## Funding

The work was accomplished working as Young Scientist, Department of Science and Technology (SYST) (Grant Number: SP/YO/2019/1385(G)). DST is highly acknowledged for providing the research grant.

## Conflict of interest

The authors declare that the research was conducted in the absence of any commercial or financial relationships that could be construed as a potential conflict of interest.

## Publisher’s note

All claims expressed in this article are solely those of the authors and do not necessarily represent those of their affiliated organizations, or those of the publisher, the editors and the reviewers. Any product that may be evaluated in this article, or claim that may be made by its manufacturer, is not guaranteed or endorsed by the publisher.
